# Improved Labeling of Pancreatic Islets Using Cationic Magnetoliposomes

**DOI:** 10.3390/jpm8010012

**Published:** 2018-03-12

**Authors:** Rita Sofia Garcia Ribeiro, Ashwini Ketkar-Atre, Ting Yin, Karim Louchami, Tom Struys, Ivo Lambrichts, Abdullah Sener, Willy Jean Malaisse, Marcel De Cuyper, Uwe Himmelreich

**Affiliations:** 1 Biomedical MRI/MoSAIC, Department of Imaging and Pathology, University of Leuven, Herestraat 49, B3000 Leuven, Belgium; Rita.Sofia.Garcia.Ribeiro@vub.ac.be (R.S.G.R.); ashket2982@gmail.com (A.K.-A.); yinting527@gmail.com (T.Y.); 2 Laboratory for Functional and Metabolic Imaging, Ecole Polytechnique Federale Lausanne, 1015 Lausanne, Switzerland; 3 Laboratory of Experimental Hormonology, ULB, Campus Erasme, Route de Lennik 808, B1070 Brussels, Belgium; karim.louchami@ulb.ac.be (K.L.); abdsener@ulb.ac.be (A.S.); 4 Laboratory of Histology, Biomedical Research Institute, Hasselt University, Campus Diepenbeek, Agoralaan, B3590 Diepenbeek, Belgium; tom.struys@sprofit.com (T.S.); ivo.lambrichts@uhasselt.be (I.L.); 5 Department of Biochemistry, ULB, Campus Erasme, Route de Lennik 808, B1070 Brussels, Belgium; malaisse@ulb.ac.be; 6 Laboratory of BioNanoColloids, Interdisciplinary Research Centre, University of Leuven, Etienne Sabbelaan 53, B8500 Kortrijk, Belgium; marcel.decuyper@kuleuven.be

**Keywords:** pancreatic islets, islet transplantation, MRI, contrast agents, magnetoliposomes, SPIOs, insulin, diabetes mellitus, cell imaging, transmission electron microscopy

## Abstract

Pancreatic islets (PIs) transplantation is an alternative approach for the treatment of severe forms of type 1 diabetes (T1D). To monitor the success of transplantation, it is desirable to follow the location of engrafted PIs non-invasively. In vivo magnetic resonance imaging (MRI) of transplanted PIs is a feasible cell tracking method; however, this requires labeling with a suitable contrast agent prior to transplantation. We have tested the feasibility of cationic magnetoliposomes (MLs), compared to commercial contrast agents (Endorem and Resovist), by labeling insulinoma cells and freshly isolated rat PIs. It was possible to incorporate Magnetic Ressonance (MR)-detectable amounts of MLs in a shorter time (4 h) when compared to Endorem and Resovist. MLs did not show negative effects on the PIs’ viability and functional parameters in vitro. Labeled islets were transplanted in the renal sub-capsular region of healthy mice. Hypointense contrast in MR images due to the labeled PIs was detected in vivo upon transplantation, while MR detection of PIs labeled with Endorem and Resovist was only possible after the addition of transfection agents. These findings indicate that MLs are suitable to image PIs, without affecting their function, which is promising for future longitudinal pre-clinical and clinical studies involving the assessment of PI transplantation.

## 1. Introduction

Type I diabetes (T1D) is a chronic disease that results from autoimmune destruction of insulin-secreting pancreatic beta cells. The subsequent lack of the insulin hormone leads to increased blood glucose levels and subsequent morbidity. In severe forms of diabetes, the current treatment of insulin administration is inadequate, and pancreatic islet (PI) transplantation is considered as a promising therapeutic alternative, as 20% of patients were insulin independent five years after PI transplantation [1]. Although the portal vein remains the most frequently used site for islet transplantation in the clinic, it is far from ideal [2,3]. Hence, many pre-clinical studies have explored renal sub-capsule, abdominal cavity, intramuscular, subcutaneous, and intraperitoneal spaces as potential alternative sites for islet transplantation [4]. Current methods of determining the islet graft function include measurements of the patient’s blood glucose, C-peptide, and glycosylated hemoglobin levels [5]. However, these methods are not sufficient to obtain an overall picture of the fate of transplanted PIs, due to the low rate of islet engraftment, islet graft rejection, recurrence of autoimmunity, and toxicity of immunosuppressive drugs [6]. Hence, the development of sensitive, non-invasive imaging methods to assess the functional status of islet grafts is of utmost importance. In a long-term perspective, in vivo imaging methods might not only provide information on the location of engrafted islets, but would potentially also allow a detailed investigation of islet graft function, in order to track changes in islet mass over time, facilitating early assessment of graft rejection and enabling earlier intervention to salvage graft function when necessary [3].

Advanced technologies for clinically applicable imaging have the potential to overcome some of the early challenges in PIs transplantation. Owing to its high resolution and excellent soft tissue contrast, magnetic resonance imaging (MRI) is the primary imaging modality utilized for cell tracking studies [7,8,9]. In order to visualize transplanted cells by MRI, the cells need to be labeled with a contrast agent prior to transplantation. Many studies have utilized superparamagnetic iron oxide particles (SPIOs), due to their high sensitivity and relatively low toxicity profile [7,8,9,10]. Earlier studies have demonstrated the feasibility to detect SPIO-labeled islets or their clusters by MRI [11,12,13,14,15,16,17].

Despite the current development of PI labeling and imaging techniques, further improvements of contrast agents used for islet labeling are necessary to enhance their viability and to allow for broad scale clinical applications. Most of the pre-clinical and initial clinical studies have utilized MR contrast agents for PI labeling that were originally approved by the Food and Drug Administration for clinical use, such as Endorem and Resovist. In these studies, SPIOs were incorporated after 24 to 72 h of incubation, using a transfection agent like poly-l-lysin (PLL) or electroporation [11,14,15,17]. Due to the relatively poor survival of isolated PIs in in vitro culture, as well as the cytotoxic effects of transfection agents and electroporation, those labeling methods reduce the yield of viable islets [17]. In addition, Endorem and Resovist were discontinued from production at the end of 2008 and are no longer commercially available [7]. Since then, new types of SPIOs have been explored, to investigate their suitability for PI labeling [18,19,20].

One promising alternative is the use of phospholipid-coated SPIOs, called magnetoliposomes (MLs). MLs consist of iron oxide cores, which are coated by a phospholipid bi-layer. The inner phospholipid layer is strongly chemisorbed onto the iron oxide core, while the outer layer is more loosely adsorbed, which allows for modulations of this outer layer by varying the content of the vesicle/acceptor ML ratio [21]. Hereby, modifications of the surface charge, incorporation of fluorescent dyes, or attachment of receptor targeting moieties are possible [22]. For cell-labeling purposes, cationic MLs based on 3.33% DSTAP (1,2-distearoyl-3-trimethylammoniumpropane) were found to be the most suitable. These MLs are nontoxic, and allow high intracellular iron concentration in a wide variety of cell types, including primary human umbilical vein endothelial cells (HUVEC), murine C17.2 neural progenitor cells (NPCs), PC12 rat pheochromocytoma cells, and mesecnchymal stem cells (MSCs) [22,23].

In this study, we have tested in vitro labeling protocols for the INS-1E cell line and freshly isolated PIs. Use of the insulinoma cell line ensured reproducibility, as cell lines are more robust than PIs, and can be cultured for multiple numbers of passages [24]. Labeling outcomes were compared with the clinically-approved iron oxide contrast agents Endorem and Resovist, in terms of labeling efficiency, toxicity, islet functionality, and detectability with MRI, both in vitro and in vivo.

## 2. Materials and Methods

### 2.1. Cell Culture, Pancreatic Islets Isolation, and Labeling

#### 2.1.1. Cell Culture

INS-1E cells were cultured in a RPMI-1640 medium (+l-Glutamine, Gibco, Ghent, Belgium) supplemented with 10% fetal bovine serum (FBS, Hyclone, Aalst, Belgium), 10 mM Hepes (Sigma, Bornem, Belgium), 1 mM sodium-pyruvate (Gibco), 0.05 mM 2-mercaptoethanol (Gibco), and 1% penicillin/streptomycin (Cellgro, Manassas, WA, USA).

#### 2.1.2. Pancreatic Islets Isolation

PIs were isolated from Sprague Dawley rats (female, 200–250 g) by the collagenase digestion method [25]. Rat donors were chosen due to simplicity of pancreas perfusion procedure and higher islet yield, compared to mouse donors. Animals were euthanized by intraperitoneal overdose of Nembutal (CERVA, Brussels, Belgium). All principles of laboratory animal care were followed according to the Guide for the care and use of laboratory animals, eighth edition (2011) and the latest European (Directive 2010/63/EU) and Belgian (Royal Decree of 29 May 2013) regulations on the protection of animals used for scientific purposes, and supervised by a qualified veterinarian. All animal experimental procedures were approved by the Ethics Committee of the KU LEUVEN (ECD number P076/2016). The pancreas was digested using 0.54 mg/mL collagenase (Roche, Vilvoorde, Belgium). Digested tissue was washed several times with cold Hanks’ Balanced Salt Solution (HBSS, Gibco) before islets were handpicked and counted. Islets were cultured in RPMI-1640 (+l-Glutamine, Gibco) medium supplemented with 10% FBS (Hyclone) and 1% penicillin/streptomycin (Cellgro).

#### 2.1.3. Contrast Agents and Labeling Experiments

Labeling experiments were performed using (1) Endorem (Guerbet, Roissy, France), (2) Resovist (Schering AG, Germany), and (3) in-house synthesized cationic MLs (Supplementary Table S1). ML synthesis and functionalization of MLs with fluorescent dye (0.75% rhodamine) was performed as described before [21,26]. INS-1E cell-labeling experiments were performed using different labeling concentrations for different incubation times (4 and 24 h). Initial experiments for cell labeling with Endorem or Resovist indicated the necessity of poly-l-lysin (388 kDa at 1.5 μg/mL), as also observed previously [17]. In contrast, labeling with cationic MLs was always performed without PLL. Labeled INS-1E and PIs were further divided for different assays, like total cell count, cell viability assay, iron quantification, and MR imaging. Unlabeled cells were always included as a negative control in all of the experiments.

Although Endorem and Resovist are no longer commercially available, they have been successfully tested in several clinical and preclinical studies for visualizing engrafted cells in general, and pancreatic islets in particular [7,8,9,10,11]. Endorem and Resovist have also been used as reference contrast agents in our laboratory for testing novel nanoparticles for cell labeling [23,27,28,29]. As a consequence of the withdrawal of Endorem and Resovist from the market, part of the batches used for the described experiments were seven years old. For this reason, regular quality control experiments were performed to confirm the integrity of these nanoparticles (see Supplementary Figure S1).

### 2.2. Uptake Confirmation

#### 2.2.1. Prussian Blue Staining

To confirm uptake of the different SPIO’s, Prussian blue staining and TEM was performed. Cells labeled for 24 h at 50 μg Fe/mL were washed three times with PBS. Labeled cells were fixed in 4% paraformaldehyde (PFA) for 15 min at room temperature; then, 2% HCl (Vel Labs, Leuven, Belgium) and 2% potassium ferrocyanide (Sigma) were mixed in equal volumes, followed by 15 min incubation at room temperature. Samples were washed three times with PBS before image acquisition.

#### 2.2.2. Cellular Iron Content

Quantification of the intracellular iron content in the cells/PIs was performed with inductively-coupled plasma optical emission spectrometry (ICP-OES, Varian720ES, Santa Clara, CA, USA). Labeled cells/PIs, subjected for 24 h to co-incubation with a 50 μg Fe/mL containing medium, were washed three times with PBS. Further INS-1E cells were trypsinized, and the cell suspension was spun down (500 g) and counted. Cell pellets of 500 cells, or 100 handpicked PIs, were lysed with concentrated HCl (3.7%). Samples were further homogenized with distilled water. Standard solutions of 0.1, 0.5, and 1.0 ppm were measured before the first sample and after every tenth sample.

#### 2.2.3. Transmission Electron Microscopy

Transmission electron microscopy (TEM) analysis was performed to determine the SPIOs’ distribution within the PIs. Approximately 25 labeled PIs were handpicked and fixed with 2% glutaraldehyde (Laborimpex, Brussels, Belgium) in a 0.05 M sodium cacodylate buffer (Aurion, Wageningen, The Netherlands) at pH = 7.3 and a temperature of 4 °C. Samples were further prepared as described by Struys et al. with minor modifications [27]. Briefly, following fixation, the PIs were dehydrated in graded concentrations of acetone and embedded in epoxy resin (araldite—Aurion, Wageningen, The Netherlands). Samples were cut into sections of 40–60 nm, using a Leica EM UC6 microtome (Leica, Groot Bijgaarden, Belgium) and transferred to 50 mesh copper grids (Aurion, Wageningen, The Netherlands) coated with 0.7% formvar. TEM analysis was performed with a Philips EM208 S electron microscope (Philips, Eindhoven, The Netherlands) operated at 80 kV, and provided with a Morada Soft Imaging System camera to acquire high resolution images of the samples. The images were processed digitally with the iTEM-FEI software (Olympus SIS, Münster, Germany).

### 2.3. Viability and Proliferation Assays

To evaluate the effect of labeling on the cells’ proliferation and viability (of INS-1E and PIs), total cell counts and Propidium iodide/Fluorescein diacetate (FDA) staining were performed, respectively. INS-1E cells were seeded and labeled with 50 μg Fe/mL medium using MLs, Endorem and Resovist (±PLL) for 24 h. After the co-incubation with the SPIOs, cells were washed three times with phosphate buffered saline (PBS without Mg^2+^ and Ca^2+^, Gibco) and incubated in an iron-free medium for six hours. Afterwards, cells were trypsinized with 0.05% trypsin (Gibco) and counted, using an automatic cell counter (Chemometec, Lillerod, Denmark). For determining their viability, labeled cells/PIs were incubated with 20 μg/mL of Propidium Iodide (Sigma), and FDA (Sigma) for 20 min at room temperature. Cells were washed with PBS, and immediately observed under a fluorescence microscope (Olympus CKX 41).

### 2.4. Glucose-Stimulated Insulin Secretion Assay

Insulin production by labeled cells/PIs was assessed by stimulating insulin secretion in the presence and absence of Theophylline in vitro. Cells were seeded and labeled with 50 μg Fe/mL medium of MLs, Endorem, Resovist (+PLL) for 24 h. Labeled cells were washed three times with PBS and incubated in an iron-free medium for 4 h. Labeled cells/PIs were incubated with basal Krebs-Ringer buffer (KRB) for 90 min at 37 °C, in the presence of either low (1.1 mM) or high (11.1 mM) concentrations of d-glucose, under constant and gentle shaking. At the end of the incubation, 500 μL of supernatant was taken out and stored at −20 °C, until determining the insulin secretion by a radioimmunoassay [30].

### 2.5. Islet Transplantation

All principles of laboratory animal care were followed according to the Guide for the care and use of laboratory animals, Eighth edition (2011) and the latest European (Directive 2010/63/EU) and Belgian (Royal Decree of 29 May 2013) regulations on the protection of animals used for scientific purposes, and supervised by a qualified veterinarian. All animal experimental procedures were approved by the Ethics Committee of the KU LEUVEN (ECD number P076/2016). C57Bl6 adult mice (*n* = 12) were used for renal capsular transplantation of pancreatic islets. Mice were anesthetized using Ketamine 1000 (CEVA) + Domitor (Janssen Pharmaceutica, Beerse, Belgium). After disinfecting the skin, a small incision was made to expose the left kidney. A total of 200 rat PIs, labeled with SPIOs (±PLL), were collected in a catheter and injected under the kidney capsule. Immediately afterwards, the wound was closed and the still anesthetized animals were subjected to MR imaging as mentioned below. To avoid early xenograft rejection, animals were provided 3 mg/kg of water-soluble dexamethasone (Merck KGaA, Darmstadt, Germany) in the drinking water from 72 h prior until the end of the experiment.

### 2.6. Magnetic Resonance Imaging

#### 2.6.1. Phantom Preparation

To test the MR detectability threshold of cells labeled under different conditions, agarose phantoms were prepared and MR imaging was performed. Hereby, labeled cells (50 μg Fe/mL of medium co-incubated for 24 h) were washed three times with PBS and trypsinized. Further, the cell suspension was spun down (1500 rpm) and counted. A total of 105 cells were again pelleted and re-suspended in 100 μL of PBS. These cell suspensions (1000 cells/μL) were mixed with 1.5% agarose (Sigma) in 1:1 ratio and transferred in 500 μL microcentrifuge tubes, 1/3 prefilled with solidified agarose. All microcentrifuge tubes (filled with 500 cells/μL) were assembled in another purpose-built plastic container filled with 1.5% agarose, according to previously described methods [28]. T2 values for the different labeling conditions were determined from multi-slice-multi-echo MRI experiments (see Supplementary Figure S2).

#### 2.6.2. Magnetic Resonance Imaging and Data Processing

All MR measurements were performed using a 9.4T Bruker Biospec small animal MR scanner (Bruker Biospin, Ettlingen, Germany; horizontal bore 20 cm), equipped with actively shielded gradients (600 mT m^−1^). In vitro and in vivo data were acquired using a quadrature radio-frequency resonator (transmit/receive; inner diameter 7 cm, Bruker Biospin). Images were processed with Paravision 5.1 (Bruker Biospin, Ettlingen, Germany). For all MRI experiments, a pilot scan was performed consisting of orthogonal slices for geometry planning of subsequent scans. For the characterization of contrast agents and labeled cells, agar phantoms were prepared as described above [23,28]. Two-dimensional multi-slice-multi-echo (MSME) scans were acquired for the calculation of T2 values (TR (repetition time) = 3000 ms and 16 TE (echo time) increments of 8 ms, with a 256 × 256 matrix conferring a 234 μm^2^ in-plane resolution). Three-dimensional, high-resolution, T2*-weighted MR images were acquired using a gradient echo sequence (Fast Low Angle Shot sequence (FLASH), TR = 200 ms, TE = 15 ms). The field of view was 6.0 × 6.0 × 2.25 cm, resulting in an isotropic resolution of 234 μm. The overall acquisition time was 2 h 10 min.

Mice were scanned in vivo under 1–2% isoflurane in O_2_. Animals were scanned on the day of the islet engraftment, and on days two and four post-islet transplantation. A respiration-gated FLASH sequence (TE = 2.3 ms, TR = 202.56 ms; six slices, with a thickness of 1 mm and an in-plane resolution of 136 μm^2^) was used to determine the decrease in the signal intensity, due to labeled islets at the site of transplantation. Mice were monitored using a monitoring and gating model (type 1030) from SA Instruments Inc. (Stony Brook, NY, USA) for controlling physiological parameters. The body temperature (using a rectal probe) and respiration rates were monitored and maintained during the acquisition at 37 ± 1 °C and 60–90 min^−1^, respectively. All in vivo MRI measurements were respiration triggered. The overall acquisition time was 23 min per animal.

### 2.7. Histology

Mouse kidneys were isolated on days two and five post-PI transplantation, fixed overnight in 10% neutral buffered formalin, and embedded in paraffin. Subsequently, 5 μm-thick sections were made. Paraffin sections were de-paraffinized and dehydrated by passing them through a graded alcohol series. To identify SPIO-containing islets, Prussian blue staining was performed. The slides were placed in a mixture containing an equal volume of 10% potassium ferrocyanide (Sigma) and 20% HCl (Vel Labs, Leuven, Belgium) for 20 min at room temperature, and then rinsed in tap water for 15 min. After 10 min of nuclear fast red counterstaining, the slides were passed through a graded series of alcohol and xylene before mounting with DPX (Sigma). Images were acquired using a Mirax Desk (Carl Zeiss, Göttingen, Germany).

### 2.8. Statistical Analysis

Statistical analysis was performed using Graphpad Prism 5 software (Graphpad, La Jolla, CA, USA). Significant differences were determined using the two-way analysis of variance (ANOVA) test with a Tukey or Bonferroni post-test. Data were plotted as mean ± standard deviation (SD), and *p*-values ≤ 0.05 were considered statistically significant.

## 3. Results

### 3.1. Labeling Optimization and Uptake Confirmation of Magnetoliposomes by INS-1E Cells

**Figure 1:**
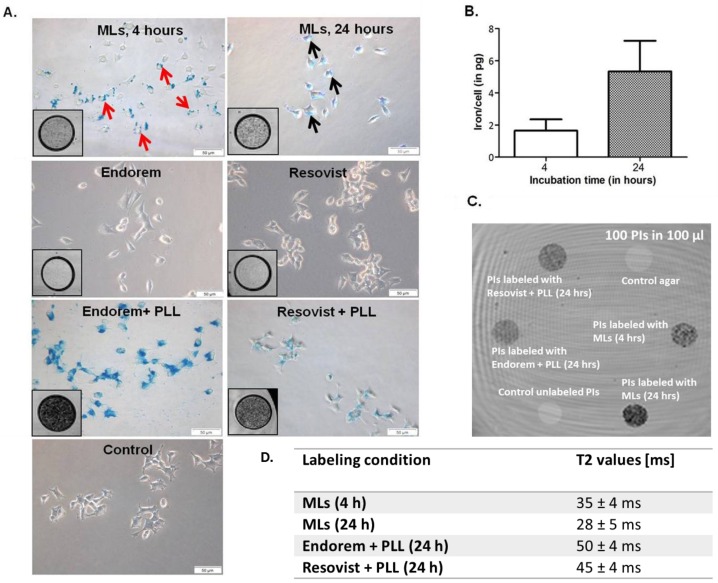
Iron uptake and confirmation of labeling: (**A**) Prussian Blue staining of INS-1E cells labeled with magnetoliposomes (MLs) at 50 μg Fe/mL medium for 4 h indicates that MLs adhered on the cell membrane (red arrows), whereas longer incubation (24 h) resulted in particle uptake and prominent blue staining in the cell cytoplasm (black arrows). Prussian Blue staining of INS-1E cells labeled with Endorem and Resovist alone at 50 μg Fe/mL medium for 24 h indicated no or only marginal uptake, whereas 100% of cells were labeled when superparamagnetic iron oxide particle (SPIOs) were conjugated with poly-l-lysin (PLL). Inserts in all Prussian blue-stained microscopic images show the T2*-weighted MR images for the different labeling conditions after 24 h of labeling. Hypointensity (dark contrast) represents the labeled cells. (**B**) Inductively-coupled plasma optical emission spectrometry (ICP-OES) measurements of INS-1E cells labeled with MLs showed a marked increase in iron uptake with increasing incubation time (1.64 ± 0.70 pg/cell at 4 h and 5.33 ± 1.9 pg/cell at 24 h) (Mean ± S.D.). Scale bar: 50 μm. (**C**) T2-weighted MR image of agar phantom-containing labeled pancreatic islets (100 islets suspended in 100 μL after labeling with Resovist + PLL, Endorem + PLL, and MLs. (**D**) T2-values acquired from the phantoms shown in (**C**).

The minimal required labeling time for cells was defined as the time required for sufficient iron accumulation in the cells that would allow reliable detection on MR images, without affecting the cells’ viability. INS-1E cells were incubated with a series of different concentrations of all the SPIOs. Figure 1A clearly shows the presence of SPIOs adhered to the INS-1E cell membranes (red arrows) after 4 h of incubation with MLs. Longer incubation (24 h) resulted in endocytosis of the MLs by the cells. The black arrows indicate the presence of blue staining, representing iron-containing endosomes. When the incubation time was increased from 4 to 24 h, the cellular uptake of MLs increased four-fold (1.64 pg/cell vs 5.33 pg/cell respectively, see Figure 1B). In contrast, incubation with the formally commercially available SPIOs Endorem and Resovist, at 50 μg Fe/mL medium for 24 h, resulted in almost no uptake (Figure 1A), except when conjugated to PLL. For INS-1E cells, when labeled with Endorem and Resovist conjugated with PLL, almost all of the cells were found to be Prussian blue-positive (at 50 μg Fe/mL medium for 24 h). T2*-weighted, high-resolution MR images (Figure 1, inserts in all Prussian blue staining images) of all labeling conditions confirmed these uptake findings. Hypointense spots representing labeled cells indicate a decrease in the signal intensity, which is proportional to the uptake. This was quantitatively confirmed by the T2 values for islets in agar phantoms under similar conditions (same iron concentration in labeling medium and same number of islets in phantoms, see Figure 1C). Therefore, these initial labeling experiments indicated the necessity for using PLL (1–5 μg/mL) when cells were labeled with Endorem and Resovist for 24 h (with 50 μg Fe/mL medium), but not for MLs.

### 3.2. In Vitro Evaluation of INS-1E Cells Post-Labeling

**Figure 2:**
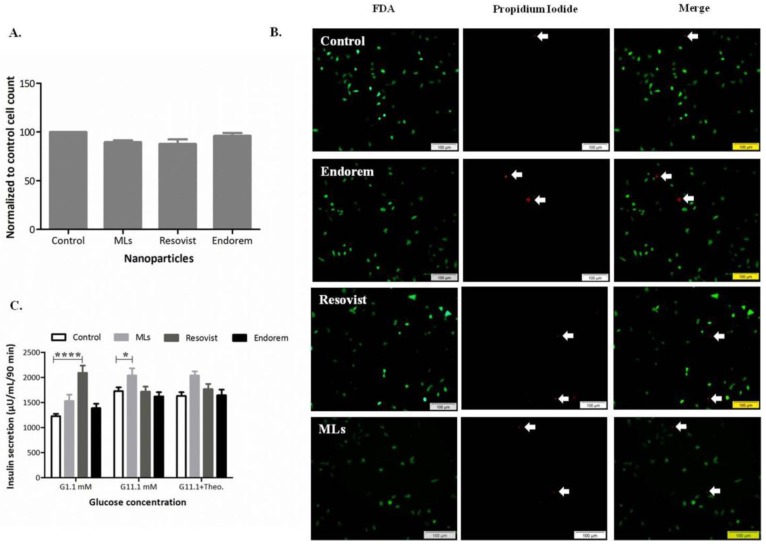
Cell proliferation and viability post labeling: (**A**,**B**) Total cell count post-labeling was comparable to non-labeled samples. (**B**) Viability with fluorescein diacetate (FDA)/Propidium Iodide staining indicated no negative effect on the cells. (**C**) d-glucose-stimulated insulin secretion did not alter when cells were labeled with Endorem. However, Resovist labeled cells at 1.1 mM and ML labeled cells at 11.1 mM glucose concentration showed an increase in the insulin secretion. Scale bar: 100 μm; data expressed in mean ± standard deviation (SD).

To assess the proliferation and viability of INS-1E cells labeled with MLs, versus commercially available SPIOs in vitro, total cell counts and FDA/Propidium iodide staining were performed, respectively. Figure 2A indicates that when INS-1E cells were labeled with three different SPIOs under the same labeling condition (50 μg Fe/mL medium for 24 h), no negative effect on cell proliferation could be observed. Total cell counts of all INS-1E cell samples were always compared with non-labeled control samples. Viability staining post-labeling, as shown in Figure 2B, indicated only a non-significant, marginal amount of dead cells (in red), compared to the viable (in green) cells observed for all labeling conditions. We further investigated whether the labeling (at 50 μg Fe/mL medium) affects the insulin secretion of INS-1E cells after stimulation with glucose at different concentrations (1.1 mM, 11.1 mM in presence and absence of theophylline). Figure 2C shows that INS-1E cells labeled with Resovist showed a significant increase in insulin secretion at hypoglycemic glucose concentration (1.1 mM). MLs-labeled INS-1E cells also showed a slight elevation in the insulin secretion at hyperglycemic (11.1 mM) glucose stimulation. Insulin secretion levels remained comparable to the values from unlabeled control cells when labeled with Endorem and stimulation in presence of theophylline.

### 3.3. In Vitro Evaluation of Pancreatic Islets Post-Labeling

**Figure 3:**
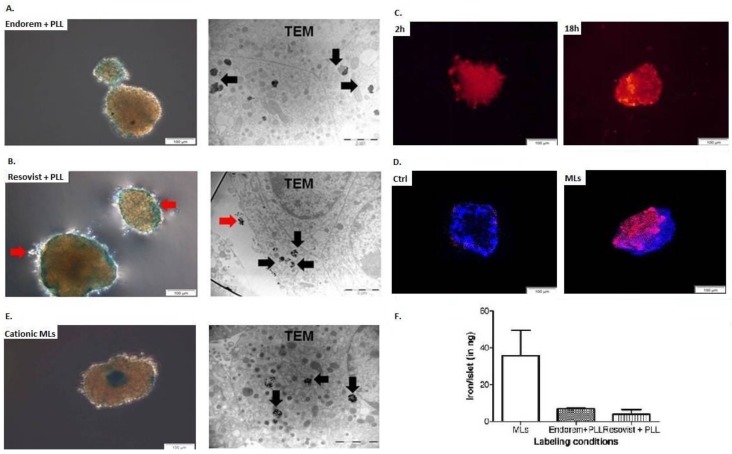
Iron uptake by rat islets: Iron uptake by pancreatic islets (PIs) was confirmed by Prussian blue staining and transmission electron microscopy (TEM), when labeled with (**A**) Endorem + PLL, (**B**) Resovist + PLL, and (**C**) cationic MLs. PIs labeled with Endorem + PLL and Resovist + PLL showed heterogeneous uptake in different islets of the same batch. MLs showed uptake already after 2 h, and elevated uptake was observed after 18 h of co-incubation with MLs. (**D**) Confocal microscopy was performed to confirm the uptake of the MLs by PIs. (**E**) Prussian blue staining and TEM were performed to confirm the uptake. The presence of particles adhered to the cell membrane is indicated by red arrows for the Prussian blue and TEM images. (**F**) Iron content in the labeled PIs (*n* = 2) was measured with inductively-coupled plasma optical emission spectrometry (ICP-OES). Scale bar: 2 μm for TEM; data expressed as mean ± SD.

We next investigated whether the optimal labeling concentration determined for INS-1E cell labeling can be translated to the labeling of freshly-isolated PIs. PIs were always labeled with 50 μg Fe/mL for 24 h with MLs, Endorem + PLL, and Resovist + PLL. Figure 3 indicates Prussian blue staining and TEM images for iron uptake confirmation after labeling. When labeled with Endorem + PLL (Figure 3A) and Resovist + PLL (Figure 3B), uptake was heterogenous throughout the islets, but the SPIOs were found to be either trapped in lysosomes (black arrows) or adherent to the cell membrane (red arrows), as confirmed by TEM. When PIs were labeled with rhodamine-positive MLs, a fluorescent signal was observed already after 2 h of incubation, but uptake was confined mainly to the peripheral cells (Figure 3C). Incubation for an additional 18 h showed homogenous uptake throughout the islet, which was also confirmed by confocal microscopy (Figure 3D), Prussian blue staining, and TEM (Figure 3E). TEM images indicated that MLs were present only in the lysosomes, and not adherent on the cell membrane or in the intracellular space of PI cells. All three nanoparticles (NPs) were found to be localized in all different subtypes of PI cells (such as β-, α-, and δ-cells), which was confirmed by the cellular characteristics identified on the ultrastructural appearance of the endocrine vesicles in TEM images. As none of the particles had an apparent specificity for particular islet cell types, the cellular uptake was most likely due to conventional fluid phase endocytosis. ICP-OES measurements of the PIs labeled with the different SPIOs have also confirmed a marked increase in the iron content for PIs when incubated with MLs at 50 μg Fe/mL medium for 24 h, when compared with PIs labeled with Endorem/Resovist + PLL.

### 3.4. Viability and Functional Evaluation of Pancreatic Islets Post-Labeling

**Figure 4:**
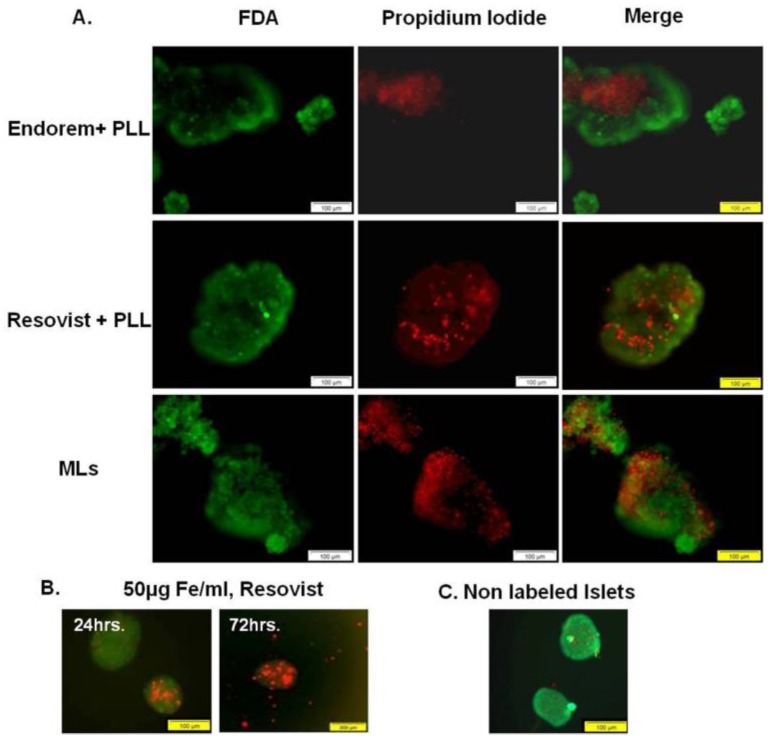
Islet viability: None of the SPIOs showed negative effects on the viability of islet cells (FDA/propidium iodide staining) after labeling. (**B**) In order to generate magnetic resonance (MR)-detectable uptake by using Resovist, without addition of PLL, 72 h of incubation was necessary. But severe effects on the islet viability were observed after such prolonged incubation times. (**C**) Non-labeled islets immediately after isolation. Scale bar: (**A**,**B**) 100 μm, (**C**) 200 μm.

To assess the viability of islets labeled with SPIOs, FDA/Propidium iodide staining was performed. Figure 4A shows fluorescent images acquired to determine the viability of PIs. PIs labeled with all three SPIOs indicated few dead cells at 24 h post-labeling. However, when PIs were labeled with Resovist (-PLL) for 72 h to achieve MR-detectable iron uptake, the viability of the islets was reduced, as seen in Figure 4B. This suggests a major influence of the incubation time on the cell viability; incubation time should therefore be kept as low as possible, in order to conserve the islet viability after SPIOs labeling. 

**Figure 5:**
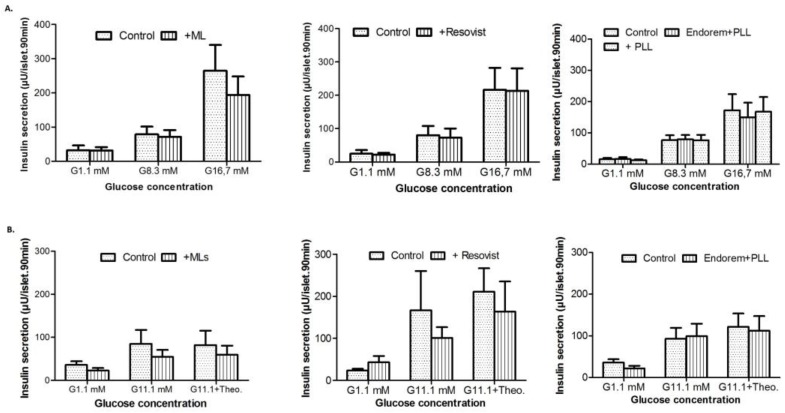
Effect of labeling PIs on their insulin secretion: (**A**) Insulin release from PIs in the presence of glucose (1.1, 8.3 and 16.7 mM) when labeled with MLs (left panel), Endorem + PLL (middle panel), and Resovist + PLL (right panel) at 50 μg/mL for 24 h. (**B**) Insulin release from islets labeled with magnetoliposomes, Endorem + PLL, and Resovist + PLL at 50 μg Fe/mL, in the presence of low (1.1 mM) and high (11.1 mM) glucose concentrations with and without 1.4 mM theophylline). These results indicate no negative effects on glucose-stimulated insulin secretion (GSIS).

To evaluate the effect of the labeling procedure on the functional capacity to secrete insulin, labeled PIs were subjected to hypo-, normo-, and hyperglycemic concentrations, using different concentrations of d-glucose. Figure 5A indicates that there was no effect observed on the insulin secretion of labeled PIs (using each of the three SPIOs) post-stimulation with d-glucose. Similar observations were made for theophylline-assisted assays, where no significant decline in the insulin secretion was observed (Figure 5B). Thus, the viability and functional assays did not show any negative effect on the PIs post-labeling, with all three SPIOs at concentrations of up to 50 μg Fe/mL medium.

### 3.5. Renal Sub-Capsular Transplantation of Pancreatic Isletss and In Vivo Magnetic Resonance Imaging

**Figure 6:**
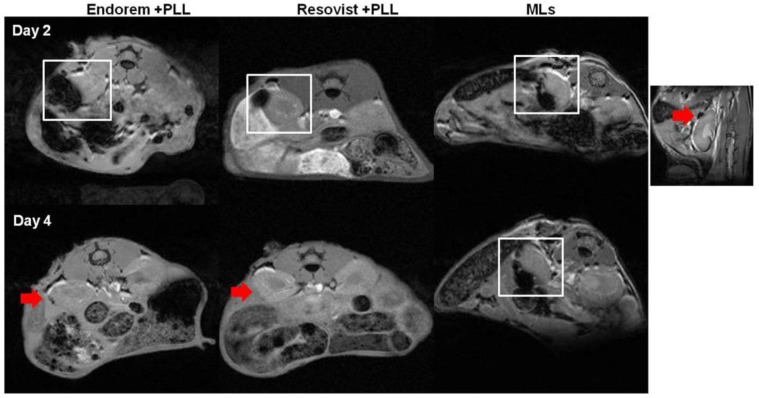
MR imaging of transplanted islets: MR imaging was performed to detect the labeled islets in vivo. The hypointense sub-capsular region (highlighted by white square) indicates PIs labeled with Endorem + PLL and Resovist + PLL. Endorem or Resovist + PLL-labeled islets were not detectable on day four in the sub-capsular region (red arrows), whereas PIs labeled with MLs were detectable until day four (indicated by white square). The right corner image indicates a prominent black spot (red arrow) seen on the sub-capsular region after transplantation of PIs labeled with MLs.

We assessed the feasibility of monitoring islets transplanted in the sub-capsular space of kidneys in healthy mice longitudinally, by using in vivo MR imaging. We implanted 200 PIs, labeled with Endorem (+PLL), Resovist (+PLL), or MLs under the left kidney capsule. Figure 6 indicates MRI performed at the different time points, indicating a marked decrease in the signal intensity (white square in Figure 6) on T2-weighted MR images. PIs labeled with MLs showed a hypointense signal until day four, whereas PIs labeled with Endorem or Resovist (+PLL) did not show any hypointense signals by day four. The higher MRI sensitivity of MLs can be explained by intracellular aggregation caused after partial degradation of the outer lipid layer in the lysosomal environment [31,32].

### 3.6. Histology

**Figure 7:**
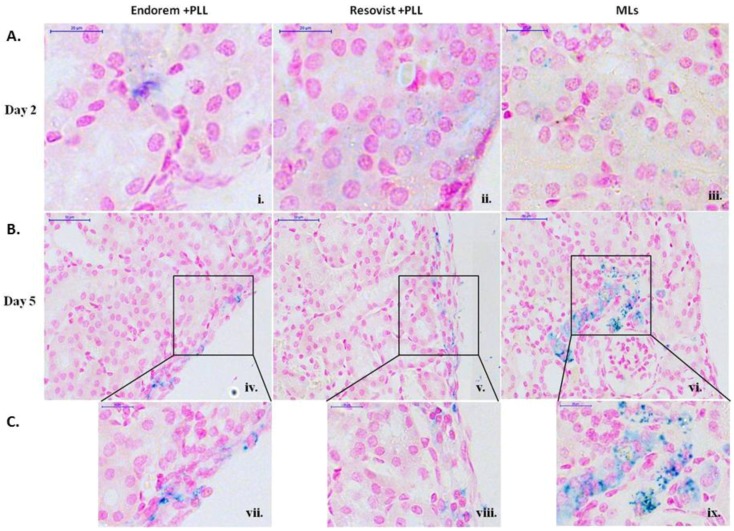
Prussian blue staining: (**A**) (**i**–**iii**) Prussian blue staining of kidney tissue clearly indicated the presence of blue stain along the sub-capsular region by day two for all labeling conditions. Figures (**B**) (**iv**–**ix**) show staining for later time points after transplantation. Prominent staining was observed for the PIs labeled with MLs for up to five days, which was not the case for the other labeling conditions. Figure (**C**) (**vii**–**ix**) shows higher magnification images for day five. Scale bar: (**i**–**iii**) and (**vi**–**ix**): 20 μm, (**iv**–**vi**): 50 μm.

In order to confirm the signal intensity reduction was indeed due to the presence of labeled PIs, Prussian blue staining of the kidney sections was performed. Figure 7 shows blue stains in the sub-capsular zone, indicating the presence of labeled islets. With histological staining, it was possible to detect labeled islets for five days post-transplantation for all examined particles. As seen from higher magnifications (Figure 7C(vii–ix)), staining pattern from islets labeled with MLs was prominently seen when compared to PIs labled with Endorem and Resovist (+PLL). This further explains the strong hypointense signal change for islets labeled with MLs and transplanted into the kidney capsules on day four.

## 4. Discussion

Food and drug administration-approved NPs like Endorem and Resovist are composed of dextran or carboxy-dextran coated iron cores, comprising multiple crystals with an overall hydrodynamic diameter of 80–120 nm and 62 nm, respectively. So far, many pre-clinical and clinical studies have indicated the feasibility of Endorem and Resovist (±PLL) to label and determine the fate of islet grafts non-invasively [11,12,13,14,15,16,17]. However, prolonged incubation times for islet labeling, and the fact that Endorem and Resovist have been withdrawn from the market, have compelled researchers to investigate alternatives for these contrast agents [11,12,14,15,16]. Numerous new SPIO contrast agents, such as Molday ION Rhodamine-BMTM (MIRB) [33], or novel dextran-coated SPIOs [33] have been studied. However, they have been mostly used for stem cell labeling or as vascular probes, and are difficult to obtain for use as reference agents [34,35].

Here, we have assessed in-house-developed, phospholipid-coated, ultra-small SPIOs called magnetoliposomes (MLs) for efficient, rapid, and biocompatible labeling and in vivo imaging of pancreatic islets, which might serve as an alternative to SPIOs when used in combination with PLL. Phospholipid bi-layers as coating material for iron oxide cores, resulting in MLs, are excellent and biocompatible MR contrast agents for cell labeling with a single iron oxide core (hydrodynamic size = 40 nm), which has been demonstrated in multiple studies [21,22]. The flexibility offered by lipid coating allows an efficient and flexible functionalization, like the ability for bimodal imaging (addition of fluorescent dye to the outer lipid layer of MLs) or the modification of different surface charges (anionic, neural, or cationic MLs) [22]. MLs have been found to be non-toxic, and allow high intracellular iron concentrations in a wide variety of cell types [22,23,36]. Thus, studying the feasibility of MLs for labeling PIs and in vivo tracking is a promising alternative to other SPIOs. Previous mechanistic studies that explained the increased relaxivity (r2/r2*) values (when compared to other SPIOs) by an intracellular clustering, after the outer phospholipid layer was removed by phospholipases, have been supported by our findings [32].

Previous studies for PI labeling with SPIOs showed heterogeneous uptake, with an overall efficiency ranging from 10–70% [12]. Therefore, we first chose to use a cell line with more uniform behavior (uptake and culture condition) for comparative uptake studies. INS-1E cells display stable differentiation into beta cell phenotypes for over 116 passages. They are able to secrete insulin in response to elevated d-glucose concentrations [23]. Insulin expression by INS-1E cells exhibits glucose concentration dependence curves similar to those of rat islets. The suitability of MLs to label INS-1E cells under different labeling conditions (0–400 μg Fe/mL, 4–24 h) was studied by using ICP-OES, and indicated an elevation in iron uptake proportional to the incubation time and concentration of MLs (Figure 1A). In contrast, no or only marginal uptake was observed when Endorem and Resovist without a transfection agent was used for labeling (Figure 1C). While the relaxation times for INS-1E cells labeled with Endoreme + PLL, Resovist + PLL, and MLs were comparable (Figure 1), MLs were more efficient than the other SPIOs + PLL for labeling PIs. In addition, the time required to incorporate a sufficient amount of MLs into PIs, in order to allow in vivo MR imaging, was only two hours (Figure 3) under culture conditions identical for the preparation of PIs for transplantation studies (37 °C, 5% CO_2_ in normal cell culture media [24]. Quantitative measurements of iron content indicated that PIs labeled with Endorem + PLL contained 6.9 ± 0.5 ng iron per islet (Figure 3F; *n* = 2), and PIs labeled with Resovist + PLL showed heterogeneous uptake resulting in 4.1 ± 2.2 ng iron per islet (Figure 3F; *n* = 2). PIs labeled with MLs took up a relatively high amount of up to 35.7 ± 13.8 ng iron per islet (Figure 3F; *n* = 2). Thus, in vitro labeling of INS-1E cells (Figure 1A) and PIs displayed a higher labeling efficiency, and therefore a higher sensitivity for their visualization under the same labeling conditions as used for labeling with Endorem and Resovist, with or without the addition of PLL. With such a high iron load, it is crucial to verify if PIs labeled with MLs retain their functional capacity to secrete insulin. In vitro experiments showed no differences in insulin secretion between labeled and unlabeled islets (Figure 5) when subjected to different glucose concentration (1.1, 8.3 and 16.7 mM) or in the presence of theophylline stimulation. Therefore, the use of cationic MLs could be useful to detect and monitor engrafted islets in experimental models of diabetes, which would mean a major step forward to follow the location of PIs after transplantation.

The main focus of this study was to determine the feasibility of MLs to label INS-1E cells and PIs efficiently, such that they are MR-detectable without exerting any toxic effects. Here, we detected 200 labeled PIs by MRI after transplantation into the renal sub-capsular region. Due to its easy access and further utilization for histological confirmations, the renal sub-capsular region is a widely used site for PI transplantation in pre-clinical rodent models. However, this model is only suitable for short-term studies, due to the poor blood supply and the subsequent oxygen-poor microenvironment, which prevents longitudinal islet survival [4]. In addition, the oxygen tension is further reduced in diabetic mice, affecting the viability of islet grafts after transplantation into diabetic mice further when compared to healthy recipients. In future studies, the clinically-used transplantation through the portal vein of the liver will be subject for longitudinal assessment. However, the benefits offered by the portal vein region in rodent models as a transplantation site would not offer similar notable benefits to the ones observed in clinical settings, due to its inaccessibility and possible surgical complications.

We have used a standard T2*-weighted MRI protocol to determine the decrease in the signal intensity in the renal sub-capsular region after engraftment of labeled islets. Due to the high iron content in islets labeled with Endorem + PLL, Resovist + PLL, or MLs, a strong signal reduction due to the “blooming effect” [8,9] was observed for all animals immediately after islet engraftment. As gradient echo sequences are more susceptible to the spatial distortions or signal loss by local field changes, compared to spin echo experiments, the exact evaluation of the contrast-enhanced region was not possible. Histological Prussian blue staining (Figure 7) confirmed the retention of MLs in the sub-capsular zone for five days post-transplantation.

## 5. Conclusions

In conclusion, we have demonstrated that PIs can be efficiently labeled by using cationic MLs, with a labeling efficiency higher than that of Endorem and Resovist. The superior characteristics of MLs (rapid labeling time of 4 h, similar contrast as with other SPIOs in the presence of PLL) highlight their potential application for islet labeling and monitoring. The results of this preliminary study are encouraging in terms of shorter incubation time (thus less stress on the islets) and no negative in vitro effect on the viability and functional properties of labeled islets. In the future, MLs should be assessed in a more clinically-relevant engraftment site, over a longer survival period of the islets. In addition, the effect of islet labeling in models of diabetes have not been assessed for MLs yet. In principle, MLs can also be further functionalized, by conjugating a functional group specific for particular receptors present on the β-cell to determine beta cell mass (BCM), in order to determine the early onset of diabetes with non-invasive imaging, similar to studies performed for liver cell targeting [36].

## Supplementary Materials

The following are available online at http://www.mdpi.com/2075-4426/8/1/12/s1. Table S1: Physico-chemical properties of superparamagnetic iron oxide MR contrast agents used in this study; Figure S1: Quality assessment for Resovist and Endorem. T2 values determined at 9.4T using in PBS; Figure S2: T2 values were determined from multi-slice-multi-echo MRI experiments for the different labeling conditions. INS-1E cells were distributed in agar at a concentration of 500 cells/ μL. All cells were labelled with 50 μg Fe/mL medium. T2 values represent mean values ± SD (*n* = 3).
