# Supplementary material for: Improved Labeling of Pancreatic Islets Using Cationic Magnetoliposomes
Source: J Pers Med. 2018 Mar 12;8(1):12. doi: 10.3390/jpm8010012 (PMC5872086; doi:10.3390/jpm8010012)
Supplement: Supplementary file 1 [file jpm-08-00012-s001.pdf]

Supplementary Materials

Table S1. Physico-chemical properties of superparamagnetic iron oxide MR contrast agents used in this study.

Name	Coating	Hydrodinamic Size (nm)	Surface Charge (mV)	$r1$ § (s^{-1}/mM)	$r2$ § (s^{-1}/mM)	Reference
Endorem	Dextran	80–150 nm	−32.24 ± 0.67	24 ± 2	98 ± 5	[1-3]
Resovist	Carboxydextran	45–62 nm	−4.4 ± 2.59	25 ± 3	151 ± 6	[1, 2, 4]
Cationic MLs	Phospholipids	40 nm	31.3 ± 7.3	15 ± 2	240 ± 8	[5-7]

§ 1.5 T, 37 °C.

Rita Sofia Garcia Ribeiro, Ashwini Ketkar-Atre, Ting Yin, Karim Louchami, Tom Struys, Ivo Lambrichts, Abdullah Sener, Willy Jean Malaisse, Marcel De Cuyper, Uwe Himmelreich

Figure S1. Quality assessment for Resovist and Endorem. T2 values determined at 9.4T using in PBS.

Contrast agent/ Date [months]	Hydrodynamic diameter [nm]	Surface charge [mV]	r_2 [s^{-1} mM^{-1}]
**Resovist**			
Time of purchase	55 ± 10	- 4.4 ± 2	155 ± 20
24 months	50 ± 8	- 5 ± 2	140 ± 15
38 months	58 ± 5	- 5 ± 1	130 ± 15
54 months	48 ± 6	- 6 ± 2	148 ± 12
68 months	52 ± 5	- 5 ± 2	119 ± 20
82 months	58 ± 5	- 4 ± 1	135 ± 15
98 months	54 ± 4	- 5 ± 1	140 ± 15
**Endorem**			
Time of purchase	120 ± 30	- 32.2 ± 1	110 ± 5
24 months	130 ± 20	- 30 ± 3	110 ± 10
38 months	100 ± 20	- 33 ± 5	95 ± 15
54 months	145 ± 20	- 30 ± 4	90 ± 18
68 months	125 ± 20	- 35 ± 4	102 ± 10
82 months	140 ± 30	- 32 ± 3	111 ± 12
98 months	130 ± 20	- 33 ± 3	100 ± 10

Figure S2. T2 values were determined from multi-slice-multi-echo MRI experiments for the different labeling conditions. INS-1E cells were distributed in agar at a concentration of 500 cells/ µL. All cells were labelled with 50 µg Fe/mL medium. T2 values represent mean values ± SD (*n*=3).

Labeling condition	T2 values [ms]
ML (4 hours)	35 ± 4 ms
ML (24 hours)	30 ± 4 ms
Endorem (24 hours)	100 ± 10 ms
Resovist (24 hours)	110 ± 15 ms
Endorem + PLL (24 hours)	18 ± 5 ms
Resovist + PLL (24 hours)	24 ± 5 ms
Unlabeled controls	140 ± 8 ms

References

1. Jung, C.W. Surface properties of superparamagnetic iron oxide MR contrast agents: Ferumoxides, ferumoxtran, ferumoxsil. *Magn. Reson. Imaging* **1995**, *13*, 675–691, doi:10.1016/0730-725X(95)00023-A.
2. Li, L.; Jiang, W.; Luo, K.; Song, H.; Lan, F.; Wu, Y.; Gu, Z. Superparamagnetic Iron Oxide Nanoparticles as MRI contrast agents for Non-invasive Stem Cell Labeling and Tracking. *Theranostics* **2013**, *3*, 595–615, doi:10.7150/thno.5366.
3. Yang, L.; Xia, Y.; Zhao, H.; Zhao, J.; Zhu, X. Magnetic resonance imaging of transplanted neural stem cells in parkinson disease rats. *J. Huazhong Univ. Sci. Technol. Med. Sci.* **2006**, *26*, 489–492, doi:10.7150/thno.5366.
4. Wang, Y.X.J. Superparamagnetic iron oxide based MRI contrast agents: Current status of clinical application. *Quant. Imaging Med. Surg.* **2011**, *1*, 35–40, doi:10.3978/j.issn.2223-4292.2011.08.03.
5. Soenen, S.J.; Vercauteren, D.; Braeckmans, K.; Noppe, W.; De Smedt, S.; De Cuyper, M. Stable long-term intracellular labelling with fluorescently tagged cationic magnetoliposomes. *Chembiochem* **2009**, *10*, 257–267, doi:10.1002/cbic.200800510.
6. Soenen, S.; Vande Velde, G.; Ketkar-Atre, A.; Himmelreich, U.; De Cuyper, M. Magnetoliposomes as MRI contrast agents. *WIREs Nanomed. Nanobiotechnol.* **2011**, *3*, 197–211, doi:10.1002/wnan.122.
7. Bulte, J.W.M.; De Cuyper, M.; Depres, D.; Frank, J.A. Preparation, relaxometry, and biokinetics of PEGylated magnetoliposomes as MR contrast agent. *J. Magn. Reson. Imaging* **1999**, *9*, 204–209, doi:10.1016/S0304-885300556-3.
